# The role of PIK3CA gene mutations in colorectal cancer and the selection of treatment strategies

**DOI:** 10.3389/fphar.2024.1494802

**Published:** 2024-10-30

**Authors:** Haitao Wang, Rui Tang, Ling Jiang, Yingtian Jia

**Affiliations:** ^1^ The School of Clinical Medical Sciences, Southwest Medical University, Luzhou, Sichuan, China; ^2^ Chengdu Anorectal Hospital, Chengdu, China; ^3^ Department of Anorectal, The Affiliated Traditional Chinese Medicine Hospital, Southwest Medical University, Luzhou, China

**Keywords:** PIK3CA gene mutation, colorectal cancer, pathogenesis, targeted therapy, immunotherapy

## Abstract

PIK3CA gene encodes the p110α catalytic subunit of PI3K, which regulates the PI3K/AKT/mTOR signaling pathway. PIK3CA gene mutation is one of the most common mutations in colorectal cancer (CRC), affecting about 15%–20% of CRC patients. PIK3CA gene mutation leads to the persistent activation of the PI3K/AKT/mTOR signaling pathway, which promotes the proliferation, invasion, metastasis, and drug resistance of CRC. This article provides a summary of the key detection methods for PIK3CA gene mutation, and provides an introduction to the existing colorectal cancer treatments and their practical applications in the clinic. Besides, this article summarizes the role and mechanism of PIK3CA gene mutation in the occurrence and development of CRC. It also explores the relationship between PIK3CA gene mutation and the clinical features and prognosis of CRC. This article focuses on the influence and mechanism of PIK3CA gene mutation on the targeted therapy and immunotherapy of CRC, and discusses the potential value and future direction of PIK3CA gene mutation in the personalized therapy of CRC. We aim to provide new perspectives and ideas for the precise diagnosis and treatment of CRC.

## 1 Introduction

Colorectal cancer (CRC) is a common malignant tumor in the digestive system (Dekker et al., 2019). In recent years, the incidence has continued to rise, posing a serious challenge to global public health. The pathogenesis of this cancer is quite complex, involving the interaction of genetic variations and environmental factors, further complicating prevention and treatment (Yan et al., 2022). Although traditional treatment methods such as surgery, radiotherapy, and chemotherapy have achieved certain efficacy (Gustavsson et al., 2015), they still face issues such as recurrence, metastasis, and drug resistance (Gupta et al., 2019; Zhou et al., 2018). Furthermore, the existence of cancer cell heterogeneity and individual differences further increases the difficulty of treatment (Amirouchene-Angelozzi et al., 2017; Graham and Sottoriva, 2017; Naxerova et al., 2017). Therefore, in-depth research on the pathogenesis of colorectal cancer and the search for more effective treatment strategies becomes particularly important.

Molecular genetics plays a crucial role in colorectal cancer research (Piawah and Venook, 2019), through the analysis of cellular genetic variations, gene mutations, epigenetic changes, and gene expression regulation (Budinská et al., 2023), revealing the complexity of the pathogenesis of colorectal cancer. This helps to identify potential pathogenic genes, driver mutations, and cancer-related signaling pathways, providing important clues for personalized treatment and the development of targeted drugs (Ciardiello et al., 2022). In addition, the research results of molecular genetics also provide theoretical support for the development of novel therapies such as microenvironment intervention and immunotherapy (Nussbaum et al., 2021; Ganesh et al., 2019). Therefore, molecular genetics is indispensable in deepening the understanding of the pathogenesis, diagnosis, and treatment of colorectal cancer. Researchers have discovered many genes related to the occurrence and development of colorectal cancer through genomic studies, including but not limited to the PIK3CA (Chang et al., 2018), KRAS (Prior et al., 2020), APC (Cen et al., 2021), SMAD4 (Zhao et al., 2018), and BRAF genes (Lan et al., 2021), among which the PIK3CA gene has attracted widespread attention. It plays a key role in the cell signaling pathway, and its mutation is closely related to the occurrence and development of colorectal cancer (Zhuang et al., 2021).

The PIK3CA gene encodes the phosphatidylinositol-3-kinase (PI3K) catalytic subunit p110α (Wu et al., 2014), and is one of the most common mutated genes in colorectal cancer, accounting for approximately 15%–20% (Rosty et al., 2013). Mutations in the PIK3CA gene can lead to sustained activation of the phosphatidylinositol 3-kinase (PI3K)/protein kinase B (AKT) signaling pathway (Chen et al., 2018), promoting the proliferation, survival, migration, invasion, angiogenesis, and drug resistance of tumor cells (Stefani et al., 2021). PIK3CA gene mutations not only affect the pathogenesis of colorectal cancer, but also correlate with the clinical characteristics, prognosis, and treatment response of colorectal cancer (Voutsadakis, 2023; Heczko et al., 2023). Therefore, in-depth study of the role and mechanism of PIK3CA gene mutations in colorectal cancer helps to reveal the molecular pathology basis of colorectal cancer and provides a basis and guidance for early diagnosis, molecular typing, risk assessment, targeted treatment, and immunotherapy of colorectal cancer.

## 2 Molecular diagnostics and therapeutic strategies for colorectal cancer

### 2.1 Detection methods for PIK3CA gene mutation

The mutation of the PIK3CA gene is closely associated with the occurrence, development, prognosis, and treatment response of colorectal cancer, making its detection clinically significant. Sanger sequencing is considered the gold standard for mutation detection due to its high accuracy. In a study involving advanced CRC patients, Sanger sequencing confirmed mutation status in conjunction with pyrosequencing, revealing a PIK3CA mutation rate of 7.5% in exon 9% and 3.6% in exon 20 (Price et al., 2015). Real-Time Fluorescence PCR offers sensitive quantification of DNA and specific mutation detection. For instance, in the context of mantle cell lymphoma, quantitative real-time PCR was effective in analyzing PIK3CA gene copy numbers, demonstrating that increased gene copies correlated with higher mRNA levels (Psyrri et al., 2009). Digital PCR, particularly multiplex drop-off digital PCR (MDO-dPCR), is a highly sensitive and precise technique for detecting mutations in key genes associated with metastatic CRC. When applied to plasma samples from 106 CRC patients, MDO-dPCR identified mutations in 42.45% of cases, achieving a sensitivity of 95.24%, a specificity of 98.53%, and an accuracy of 96.98% (Yu et al., 2023). Fluorescence *In Situ* Hybridization (FISH) enables direct visualization of gene amplification within tumor samples. A study utilizing FISH identified PIK3CA amplification in 38% of colorectal cancer sample (Jehan et al., 2009). Immunohistochemistry, on the other hand, detects protein expression related to PIK3CA mutations, providing functional insights into tumor biology. Although one study revealed no mutations in specific exons, it noted the activation of the PI3K/AKT/mTOR pathway, highlighting its role in tumorigenesis (Tasioudi et al., 2015). Finally, high-throughput sequencing allows for comprehensive mutation profiling across multiple genes. A particular study emphasized its utility in assessing mutations in the PIK3CA gene alongside others in the MAPK pathway, addressing quality control in detecting coexisting mutations and their potential therapeutic implications (Zheng et al., 2019).

### 2.2 Current therapeutic interventions in colorectal cancer

#### 2.2.1 Surgical interventions

Surgical resection remains the cornerstone of treatment for early-stage and localized CRC (Li et al., 2023a). The standard procedures include segmental colectomy or low anterior resection, depending on tumor location (Khan et al., 2017; Ammendola et al., 2023). These methods aim to remove the tumor and prevent recurrence while preserving as much of the healthy colon as possible. Surgical treatment is particularly effective for patients whose cancer has not yet spread beyond the colon. Research has indicated that the location and extent of the resection can influence outcomes. For example, a meta-analysis found that patients with T1 CRC were at higher risk of lymph node metastasis and recurrence after local resection (LR) (Chen et al., 2023).

#### 2.2.2 Chemotherapy

Chemotherapy remains an effective treatment for CRC, especially advanced CRC. The standard chemotherapeutic agents for CRC often include combinations of fluorouracil, leucovorin, and oxaliplatin (FOLFOX) (Akdeniz et al., 2021), as well as irinotecan-based regimens like FOLFIRI (Kopetz et al., 2019). These treatments help reduce tumor size, delay disease progression, and improve survival rates. However, chemotherapy is associated with significant side effects, such as nausea, vomiting, kidney injury, and pain (Ozkan et al., 2023). And its efficacy can vary depending on genetic mutations within the tumor, such as KRAS and PIK3CA mutations, which may influence resistance to certain drugs (Wang et al., 2018a).

#### 2.2.3 Targeted therapy

Targeted therapy focuses on specific molecular pathways involved in CRC. A major focus is on targeting the Epidermal Growth Factor Receptor (EGFR) pathway, particularly in patients with wild-type KRAS and NRAS genes (Wingert et al., 2021). Anti-EGFR monoclonal antibodies such as cetuximab and panitumumab are commonly used, often in combination with chemotherapy (Kopetz et al., 2021). However, tumors harboring PIK3CA mutations, especially those in exon 20, often exhibit resistance to anti-EGFR therapies, limiting their effectiveness (Fu et al., 2021). Additionally, mutations in the BRAF gene further complicate responses to targeted therapies, making combination treatments essential (Grothey et al., 2021).

#### 2.2.4 Immunotherapy

Immunotherapy, specifically immune checkpoint inhibitors like anti-PD-1 and anti-PD-L1 antibodies, has become a promising treatment for microsatellite instability-high (MSI-H) CRC or those with mismatch repair deficiencies (dMMR) (Andre et al., 2021; André et al., 2020). Immunotherapy enhances the immune system’s ability to target and destroy cancer cells by blocking inhibitory pathways. PIK3CA mutations have been linked to increased expression of PD-L1 in some cases, potentially enhancing the effectiveness of immune checkpoint inhibitors, Peng et al.'s study proves this conclusion (Peng et al., 2024). This correlation suggests that immunotherapy may be particularly effective in PIK3CA-mutated cancers, although more research is needed to fully understand this interaction.

#### 2.2.5 Radiation therapy

Radiation therapy is primarily used as an adjunct treatment in CRC, particularly for rectal cancer (Fokas et al., 2022). It can be applied before surgery (neoadjuvant) (Boublikova et al., 2023) to shrink the tumor and improve the likelihood of successful resection or after surgery (adjuvant) (Huang et al., 2024) to eliminate residual cancer cells. While radiation therapy is less commonly used in colon cancer, it plays a significant role in managing rectal cancers where precise targeting can minimize the risk of local recurrence. The integration of radiation with chemotherapy, known as chemoradiation, has proven effective in certain stages of rectal cancer, improving overall outcomes (Li et al., 2023b).

## 3 Role and mechanism of PIK3CA gene mutation in the occurrence and development of colorectal cancer

### 3.1 Types of PIK3CA gene mutations

Mutations in the PIK3CA gene have been associated with several clinical and pathological features in CRC, such as age of onset, tumor location, histological grading, and microsatellite instability status (Jin et al., 2020). The majority of PIK3CA mutations are concentrated in exons 9 and 20, which encode the helical and kinase domains of the p110α catalytic subunit of PI3K, respectively (Nosho et al., 2008; Velho et al., 2005). Recent data indicate that the most frequent PIK3CA variants observed in metastatic CRC include H1047R (9.8%), E545K (9.2%) and E542K (9.0%) (Yasin et al., 2024). However, additional mutations in other exons have been identified, albeit less frequently, and may also contribute to tumor progression and therapeutic resistance. These mutations can be broadly categorized into two main groups: helical domain mutations and kinase domain mutations, each of which plays distinct roles in CRC pathogenesis and response to therapy.

Helical domain mutations are the most common type of PIK3CA gene mutations, mainly including E542K, E545K, and Q546R (Reinhardt et al., 2022; Yeh et al., 2019) These mutations result in conformational changes in the PI3K enzyme, enhancing its ability to interact with membrane phospholipids and consequently increasing its catalytic activity (Ikenoue et al., 2005; Gymnopoulos et al., 2007). Therefore, helical domain mutations can lead to abnormal activation of the PI3K/AKT signaling pathway at multiple levels, promoting the proliferation, survival, migration, and invasion of colorectal cancer cells (Stefani et al., 2021). Notably, these mutations disrupt the normal regulatory mechanisms of PI3K, causing excessive signaling that drives cancer cell growth. Additionally, helical domain mutations are often associated with early-stage colorectal cancers, but they have also been linked to tumor resistance to certain therapies, such as anti-EGFR monoclonal antibodies, due to the persistent activation of downstream signaling pathways (Fu et al., 2021).

**FIGURE 1:**
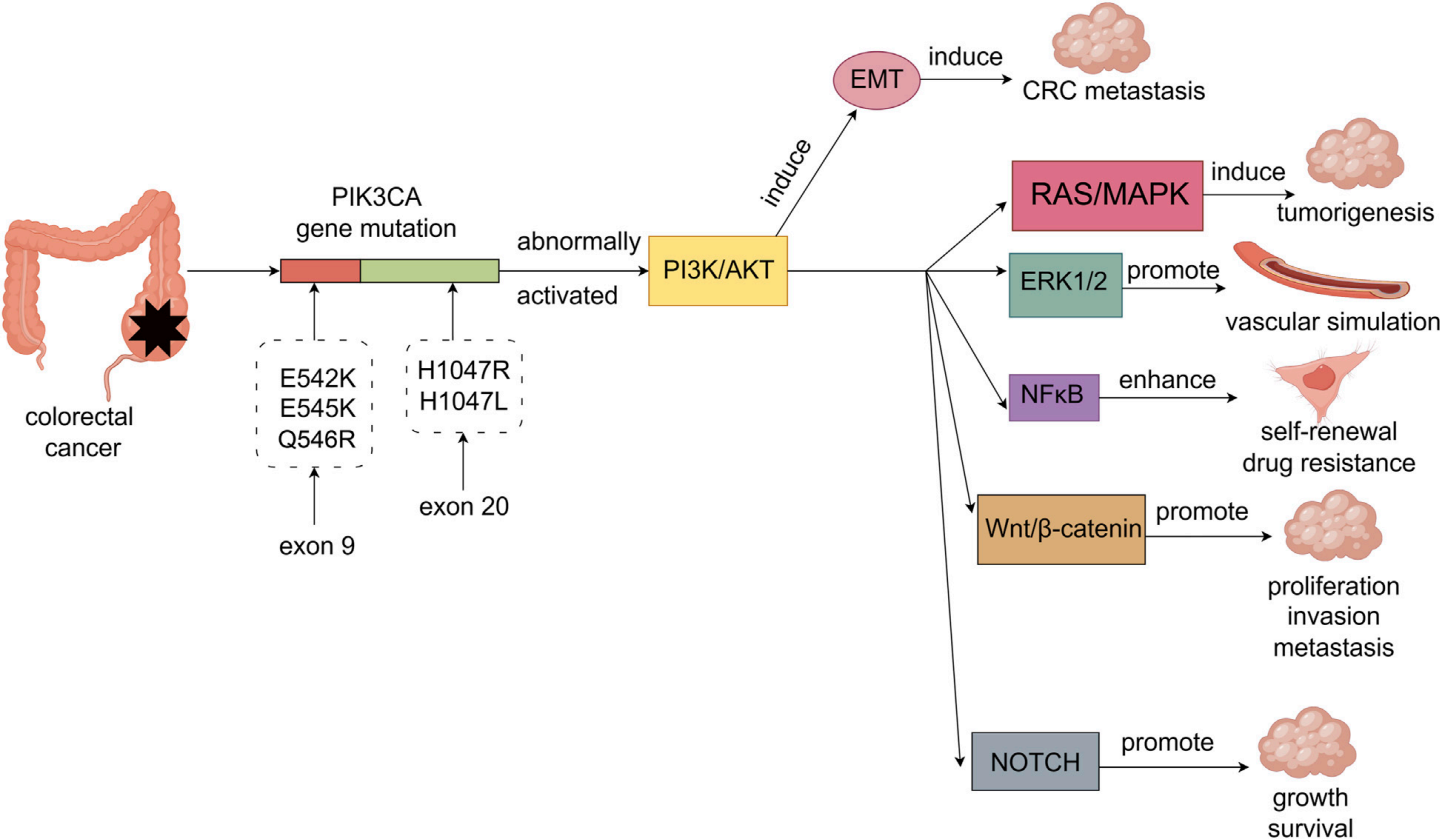
PIK3CA gene mutations in colorectal cancer are mainly E542K, E545K and Q546R on exon 9 and H1047R and H1047L on exon 20. PIK3CA gene mutations abnormally activate the PI3K/AKT signaling pathway, thereby causing EMT-induced metastasis of colorectal cancer. The PI3K/AKT signaling pathway can also crosstalk with other cancer-related signaling pathways through multiple mechanisms, thereby synergistically promoting the malignant phenotype of colorectal cancer. For example, the interaction with RAS/MAPK signaling pathway is involved in tumorigenesis. The interaction with ERK1/2 signaling pathway promotes the formation of vascular simulation in colorectal cancer cells. Interaction with NFκB signaling pathway enhances self-renewal and drug resistance of colorectal cancer stem cells. The interaction with Wnt/β-catenin signaling pathway promotes the proliferation, invasion and metastasis of colorectal cancer cells. Interaction with the NOTCH signaling pathway promotes the growth and survival of colorectal cancer cells.

Kinase domain mutations are the second most common type of PIK3CA gene mutations, mainly including H1047R and H1047L (Keppler-Noreuil et al., 2015). These mutations result in increased lipid kinase activity of PI3K, which directly amplifies the activation of downstream effector molecules, including AKT and mTOR (57). This enhanced kinase activity contributes to the abnormal and sustained activation of the PI3K/AKT/mTOR pathway, which plays a critical role in promoting cancer cell proliferation, survival, metabolic reprogramming, and angiogenesis (Canaud et al., 2021). Unlike helical domain mutations, kinase domain mutations tend to be associated with more advanced stages of CRC and are often correlated with worse prognosis (Fariña Sarasqueta et al., 2011) (Figure 1).

Beyond the well-characterized mutations in the helical and kinase domains, rare mutations such as W780 and Q859 have been reported (Varkaris et al., 2024). Although less frequent, these mutations can significantly impact CRC progression by influencing the tumor’s response to PI3K inhibitors and other targeted therapies. For example, W780 mutations have been implicated in resistance to certain PI3K inhibitors, complicating treatment strategies (Varkaris et al., 2024). Additionally, double mutations, such as those found in cis configurations (two mutations on the same allele), have been observed in some CRC cases, leading to an even more pronounced activation of the PI3K pathway (Vasan et al., 2019).

### 3.2 Molecular mechanisms of PIK3CA gene mutation in the occurrence and development of colorectal cancer

PI3K is an intracellular lipid kinase that is involved in the regulation of cell proliferation, differentiation, and survival (Narayanankutty, 2019; Moafian et al., 2021). PIK3CA gene mutations activate the PI3K/AKT signaling pathway, promoting the occurrence, metastasis, and angiogenesis of colorectal cancer (Duan et al., 2018). Furthermore, the PI3K/AKT signaling pathway can induce epithelial-mesenchymal transition (EMT) by downregulating epithelial markers while upregulating mesenchymal markers and EMT-specific transcription factors, thereby promoting the metastasis of colorectal cancer (Maharati and Moghbeli, 2023). The PI3K/AKT signaling pathway not only plays a crucial role in the occurrence and progression of colorectal cancer but also interacts with other cancer-related signaling pathways, promoting the malignant phenotype through various mechanisms. In particular, the PI3K/AKT/mTOR and Ras/MAPK pathways are frequently activated by mutations or chromosomal translocations in receptor tyrosine kinases (RTKs) such as c-Kit, PDGFR, and FLT3, which are known to be involved in oncogenesis (Matsumura et al., 2008). Chromosomal translocations often generate fusion proteins that lead to constitutive activation of these RTKs (Matsumura et al., 2008), driving continuous signaling through downstream pathways, including PI3K/AKT and Ras/MAPK(Tsai et al., 2021; Soleimani et al., 2019). Specifically, phosphorylation of downstream effectors, such as RAF, MEK, and ERK, results in enhanced cell proliferation, survival, and tumorigenesis in colorectal cancer. In this crosstalk, phosphorylated Ras can also activate PI3K (Kurig et al., 2009), forming a positive feedback loop that enhances both pathways’ activities, thereby supporting aggressive tumor growth. The crosstalk between the PI3K/AKT and ERK1/2 pathways in colorectal cancer cells is crucial in regulating vascular mimicry. This crosstalk is mediated by N6-methyladenosine (m6A) modifications, which regulate the stability and translation of mRNAs, such as EphA2 and VEGFA, which are key players in angiogenesis (Liu et al., 2022a). In addition, phosphorylation of ERK by upstream kinases in the Ras/MAPK pathway can enhance the transcription of pro-angiogenic genes, leading to the formation of new vasculature that supports tumor growth (He et al., 2023). The PI3K/AKT pathway activates NFκB signaling through phosphorylation of IκB kinase (IKK), which leads to IκB degradation and allows NFκB to translocate to the nucleus, where it activates genes involved in inflammation and survival (Shankar et al., 2019; Qi et al., 2024). Recent evidence has shown that GLI1, a key effector of the Hedgehog (HH) pathway, is closely linked to PI3K/AKT/NFκB signaling in colorectal adenocarcinoma (Yang et al., 2018). Specifically, AKT phosphorylation promotes GLI1 activation, which in turn enhances NFκB activity. This cross-talk promotes the expression of cancer stem-like cell markers (e.g., SOX9, CD133) and EMT-related genes, enhancing the malignant phenotype. Additionally, the interaction between the PI3K/AKT and Wnt/β-catenin pathways involves the direct phosphorylation of β-catenin by AKT. Phosphorylation of β-catenin results in its stabilization, preventing proteasomal degradation, and promoting its nuclear translocation (Prossomariti et al., 2020). Once in the nucleus, β-catenin interacts with transcription factors to activate Wnt target genes that drive cell proliferation, invasion, and metastasis (Prossomariti et al., 2020). This phosphorylation event is a crucial regulatory mechanism that links PI3K/AKT signaling to the activation of the Wnt/β-catenin pathway, enhancing metastatic capabilities in colorectal cancer cells. Furthermore, the PI3K/AKT pathway also regulates the NOTCH signaling pathway through inhibition of glycogen synthase kinase-3 (GSK-3) (Bertrand, 2020). GSK-3 typically phosphorylates NOTCH, marking it for degradation (Espinosa et al., 2003). AKT phosphorylates and inactivates GSK-3, preventing it from phosphorylating NOTCH. This inhibition stabilizes NOTCH, allowing for its accumulation and sustained activation of NOTCH signaling, which promotes colorectal cancer cell growth and survival. This interaction is tightly regulated through multiple layers of phosphorylation, ensuring precise control over NOTCH activity and thus contributing to the maintenance of cancer cell proliferation and survival (Figure 1).

### 3.3 The impact of PIK3CA gene mutations on the biological behavior of colorectal cancer cells

PIK3CA gene mutations have various impacts on the biological behavior of colorectal cancer cells. For example, PIK3CA mutations can affect cell proliferation through the PI3K-MEK/PDK1-GPT2 pathway regulation (Chen et al., 2022a). In addition, PIK3CA mutation-induced PI3K/Akt activation contributes to the survival and proliferation of colorectal cancer stem cells, further leading to chemoresistance (Wang et al., 2018a), and promoting epithelial-mesenchymal transition by regulating AKT activity (Miller et al., 2020). It has been found that high PI3K expression and PIK3CA mutations are associated with the metastasis of colorectal cancer cells (Zhu et al., 2012), which has been confirmed by other studies as well (Huang et al., 2018). Recent studies have shown that PIK3CA mutations in colorectal cancer led to significant metabolic reprogramming. Specifically, Hao et al. (2016) demonstrated that mutant PIK3CA upregulates the enzyme glutamate pyruvate transaminase 2 (GPT2), increasing glutamine utilization to support tumor growth. Additionally, a novel mechanism involving the nuclear translocation of the p85β subunit has been identified in PIK3CA helical domain mutations, this translocation stabilizes EZH1 and EZH2, promoting oncogenesis through enhanced chromatin modification (Hao et al., 2022). Lastly, oncogenic PIK3CA mutations reduce apoptosis and increase tumor invasion by activating the AKT signaling pathway, thereby promoting the occurrence of colorectal tumors (Whitehall et al., 2012). However, these effects are not absolute and are also influenced by other factors such as mutation frequency, level, subclonal distribution, heterogeneity, homozygosity, background mutations, etc. PIK3CA mutations also result in changes in epigenetic regulation, leading to mutation-related phenotypic heterogeneity, which may further impact the autophagy, angiogenesis, migration, invasion, and immune escape of colorectal cancer cells (Ghodsinia et al., 2020).

## 4 Relationship between PIK3CA gene mutations and clinical characteristics and prognosis of colorectal cancer

The impact of PIK3CA gene mutations on the clinical characteristics and prognosis of colorectal cancer is not fully understood, and there are certain differences and contradictions in the results of different studies. This section will review the relevance of PIK3CA gene mutations to the clinical and pathological characteristics of colorectal cancer, as well as the impact of PIK3CA gene mutations on the prognosis of colorectal cancer patients and possible mechanisms.

### 4.1 Relevance of PIK3CA gene mutations to the clinical and pathological characteristics of colorectal cancer

The relevance of PIK3CA gene mutations to the clinical and pathological characteristics of colorectal cancer, including tumor location, differentiation degree, pathological staging, lymph node metastasis, tumor metastasis, microsatellite instability (MSI), histological type, *etc.*, currently has no unified conclusion (Jin et al., 2020). Some studies have found that PIK3CA gene mutations are associated with right-sided colon cancer (Salem et al., 2020; Ye et al., 2020), poorly differentiated cancer (Zeng et al., 2023), advanced pathological staging (Chen et al., 2022b), lymph node metastasis (Byun et al., 2023), metastasis (Mao et al., 2015), microsatellite stable (MSS) (Fu et al., 2021), mucinous adenocarcinoma (Li et al., 2021), and other clinical characteristics, suggesting that PIK3CA gene mutations may be related to the malignancy and progression of colorectal cancer. However, some studies have not found a significant correlation between PIK3CA gene mutations and these clinical characteristics, and even some studies have found that PIK3CA gene mutations are associated with well-differentiated cancer (Ye et al., 2020), microsatellite instability-high (MSI-H) (Escobar et al., 2020), squamous cell carcinoma (Astaras et al., 2023), and other clinical characteristics, suggesting that PIK3CA gene mutations may be related to the benign nature and prognosis of colorectal cancer. The differences and contradictions in these research results may be related to factors such as the selection of research subjects, sample size, methods and standards of mutation detection, and methods and standards of statistical analysis. In addition, different types and locations of PIK3CA gene mutations may also have different effects on the clinical characteristics of colorectal cancer. For example, some studies have found that mutations in exon 9 are associated with right-sided colon cancer (Fu et al., 2021), well-differentiated tumors (Fu et al., 2021), MSS, etc., mutations in exon 21 are significantly associated with MSI-H status and poor differentiation (Hechtman et al., 2015), and mutations in exon 20 are associated with right-sided colon cancer (Fu et al., 2021), MSI-H (Day et al., 2013), and other clinical characteristics. Therefore, the relevance of PIK3CA gene mutations to the clinical characteristics of colorectal cancer requires further research and validation to clarify the impact of different mutation types and locations on the clinical characteristics of colorectal cancer.

### 4.2 Impact and potential mechanisms of PIK3CA gene mutations on the prognosis of colorectal cancer patients

The impact of PIK3CA gene mutations on the prognosis of colorectal cancer patients has been a subject of debate, with conflicting views and results. Some studies suggest that PIK3CA gene mutations are a negative prognostic factor for colorectal cancer patients, associated with lower overall survival (OS) and disease-free survival (DFS). This may be related to the sustained activation of the PI3K/AKT signaling pathway caused by PIK3CA gene mutations, which promotes tumor cell proliferation, survival, migration, and angiogenesis. For example, one study indicated that patients with stage IV carrying exon 20 mutations had significantly shorter OS compared to wild-type patients, and in a multivariate COX regression model, PIK3CA exon 20 mutations were significantly associated with reduced OS in stage IV colorectal cancer patients (Fu et al., 2021). Another study showed that in colorectal cancer, PIK3CA gene mutations were associated with worse OS and DFS, but not with the risk of recurrence (RR) and risk of death (HR) (Mei et al., 2016). Another study utilized circulating tumor DNA (ctDNA) detection technology and found that the detection of PIK3CA gene mutations in ctDNA was associated with worse OS and PFS, independent of treatment regimens (Dumbrava et al., 2021). Colorectal cancers with mutations in the PIK3CA gene also tend to be accompanied by mutations in other oncogenes, such as KRAS, BRAF and TP53, as well as higher tumor mutational load (TMB), which may lead to a more malignant and complex biological behavior of the tumor (Zhuang et al., 2021; Wang and Pan, 2022). For example, the prognostic value of PIK3CA mutations is related to the coexistence of KRAS mutations. double mutations in KRAS and PIK3CA are associated with poorer OS and DFS, whereas PIK3CA mutations alone are associated with better OS and DFS(99). However, some studies argue that PIK3CA gene mutations do not have a significant impact on the prognosis of colorectal cancer patients, which may be related to the interaction of PIK3CA gene mutations with other gene mutations or expressions, as well as individual patient differences (Eklöf et al., 2013; Mouradov et al., 2013). It is worth noting that different types and locations of PIK3CA gene mutations may also have different impacts on the prognosis of colorectal cancer patients. For example, a study found that mutations in exon 9 were associated with lower OS and DFS, while mutations in exon 20 were associated with higher OS and DFS(99). Therefore, the prognosis and survival value of PIK3CA gene mutations in colorectal cancer are influenced by multiple factors, including the detection method of mutations, the types and locations of mutations, the coexistence of mutations, and the clinical characteristics of patients.

## 5 Impact and mechanism of PIK3CA gene mutations on targeted therapy and immunotherapy in colorectal cancer

### 5.1 Impact and mechanism of PIK3CA gene mutations on targeted therapy in colorectal cancer

**FIGURE 2:**
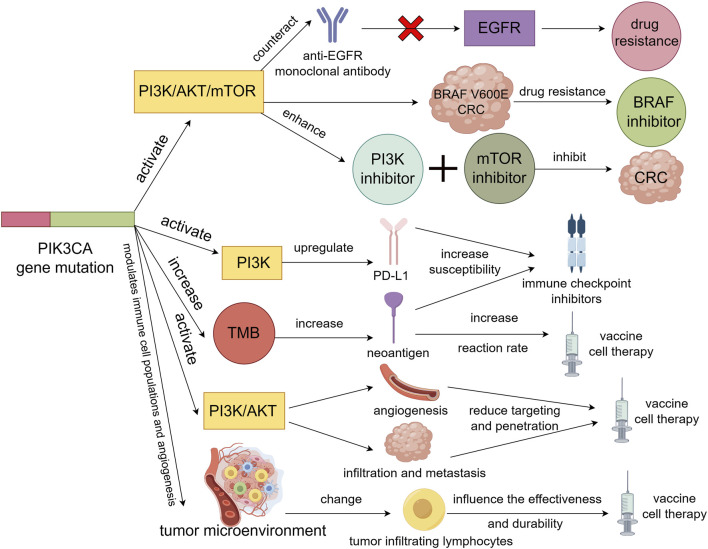
PIK3CA gene mutation activation of PI3K/AKT/mTOR signaling pathway will resist the action of anti-EGFR monoclonal antibody on EGFR signaling pathway, resulting in resistance to EGFR monoclonal antibody in colorectal cancer patients. Also causes resistance to BRAF inhibitors in BRAF V600E colorectal cancer. However, activation of the PI3K/AKT/mTOR signaling pathway may enhance the sensitivity of colorectal cancer to PI3K inhibitors and mTOR inhibitors, thereby inhibiting the tumor. Secondly, PIK3CA gene mutations not only upregulate the expression of PD-L1 by activating the PI3K signaling pathway, but also increase TMB to produce more neoantigens, which will increase the sensitivity of colorectal cancer to immune checkpoint inhibitors. Finally, PIK3CA gene mutation may promote tumor angiogenesis and tumor cell infiltration and metastasis by activating PI3K/AKT signaling pathway, thus reducing the targeting and penetration of vaccine or cell therapy. It is also possible to alter the number and activity of tumor-infiltrating lymphocytes by influencing the tumor microenvironment, thereby affecting the effectiveness and durability of vaccines or cell therapies.

Targeted therapy for colorectal cancer mainly focuses on the EGFR and VEGF signaling pathways. Currently approved drugs include anti-EGFR monoclonal antibodies (cetuximab and panitumumab) (Qin et al., 2018; Watanabe et al., 2023) and anti-VEGF monoclonal antibodies (bevacizumab and ramucirumab) (Prager et al., 2023; Hochster et al., 2024). In addition, small molecule inhibitors targeting BRAF, MEK, PI3K, and mTOR are being studied in clinical trials (Xie et al., 2020). The sensitivity and resistance of PIK3CA gene mutations to these targeted drugs vary depending on the drug and mutation type, which will be discussed separately below (Figure 2).

#### 5.1.1 Anti-EGFR monoclonal antibodies

Anti-EGFR monoclonal antibodies are an important means of targeted therapy for colorectal cancer, mainly used in combination with chemotherapy for patients with wild-type KRAS and NRAS advanced colorectal cancer, which can significantly improve the objective response rate and progression-free survival of patients (Biller and Schrag, 2021). However, there are still some patients who do not respond to anti-EGFR monoclonal antibodies or develop secondary resistance, which may be related to the activation of other signaling pathways in the tumor (Biller and Schrag, 2021).

Some studies have found that colorectal cancer patients with PIK3CA gene mutations have poor efficacy when using anti-EGFR monoclonal antibodies, with significantly shortened progression-free survival and overall survival (Wang et al., 2022; Tan et al., 2022). This may be because PIK3CA gene mutations lead to sustained activation of the PI3K/AKT/mTOR signaling pathway, counteracting the inhibitory effect of anti-EGFR monoclonal antibodies on the EGFR signaling pathway (Koustas et al., 2017). However, some studies have failed to confirm the correlation between PIK3CA gene mutations and the efficacy of anti-EGFR monoclonal antibodies, which may be related to factors such as sample size, design, and analytical methods of the study (Parseghian et al., 2019).

It is worth noting that different types and locations of PIK3CA gene mutations may have different effects on the sensitivity of anti-EGFR monoclonal antibodies. Some studies have found that only the H1047R mutation on exon 20 is associated with resistance to anti-EGFR monoclonal antibodies, while the E542K and E545K mutations on exon 9 do not affect efficacy (Li et al., 2016; Papadatos-Pastos et al., 2015). This may be because mutations on exon 20 more strongly activate the PI3K/AKT signaling pathway than mutations on exon 9 (115). Furthermore, there are also studies that have found that when PIK3CA gene mutations coexist with KRAS and BRAF mutations, the resistance to anti-EGFR monoclonal antibodies is more pronounced (Rachiglio et al., 2019). This suggests that the concurrent abnormal activation of multiple signaling pathways may collectively lead to the failure of anti-EGFR monoclonal antibodies.

#### 5.1.2 BRAF inhibitors

The BRAF gene is an important component of the RAS/RAF/MEK/ERK signaling pathway, which plays a role in processes such as cell proliferation, differentiation, and apoptosis (Ciombor et al., 2022). Mutations in the BRAF gene account for approximately 12% of metastatic colorectal cancer, with the majority being the V600E mutation (Yaeger et al., 2018). Patients with BRAF gene mutations in colorectal cancer have a poor prognosis and a low response to chemotherapy and targeted therapy (Morris and Bekaii-Saab, 2020). Currently, several small molecule inhibitors targeting BRAF gene mutations have been developed, such as vemurafenib, dabrafenib, and trametinib (Nalli et al., 2021).

Mao et al. (2013) found that PIK3CA mutations leading to activation of the PI3K/AKT pathway resulted in greater resistance to BRAF inhibitors in BRAF V600E colorectal cancer. This may be because cancer cells are able to maintain cell survival and proliferation through the PI3K/AKT pathway even when treated with BRAF inhibitors. This study also showed that synergistic inhibition of BRAF and PI3K suppressed the growth of colon cancer cells (Mao et al., 2013). Therefore, it is recommended to use combination approaches to improve the prognosis of patients with BRAF V600E colorectal cancer.

#### 5.1.3 MEK inhibitors

MEK is a downstream molecule of the RAS/RAF/MEK/ERK signaling pathway, which plays an important role in regulating processes such as cell proliferation, differentiation, and apoptosis (Ros et al., 2021). MEK inhibitors are a class of small molecule drugs targeting the MEK molecule, and several MEK inhibitors such as refametinib, binimetinib, and selumetinib are currently being studied in clinical trials (Nalli et al., 2021). MEK inhibitors are mainly used in BRAF mutant colorectal cancer patients in combination with BRAF inhibitors or other drugs to enhance efficacy and delay resistance (Ros et al., 2021; Sullivan et al., 2015).

Wee et al. (2009) found that activating mutations in PIK3CA reduced sensitivity to MEK inhibition, while downregulation of PIK3CA restored sensitivity to MEK inhibition in cells with concurrent KRAS and PIK3CA mutations. PIK3CA mutations are also associated with resistance to MEK inhibition in KRAS-mutant CRC cells (Halilovic et al., 2010; Jing et al., 2012). It has also been shown that dual blockade of the MAPK and PI3K pathways can overcome intrinsic resistance to MEK inhibition (Martinelli et al., 2013). Therefore, simultaneously inhibiting the MEK and PI3K pathways is more beneficial for tumor suppression (Temraz et al., 2015).

#### 5.1.4 PI3K inhibitors and mTOR inhibitors

PI3K inhibitors are a class of small molecule drugs that target the PI3K enzyme, aiming to directly inhibit the abnormal activation of the PI3K/AKT signaling pathway caused by mutations in the PIK3CA gene, thereby inhibiting tumor growth and metastasis. Currently, some PI3K inhibitors such as pictilisib, alpelisib, and buparlisib are being studied in clinical trials (Belli et al., 2019; André et al., 2019; Bardia et al., 2020). PI3K inhibitors are mainly used in colorectal cancer patients with PIK3CA mutations, in combination with chemotherapy or other drugs, to improve efficacy and overcome resistance (Temraz et al., 2015; García-García et al., 2015). Colorectal cancer with PIK3CA mutations often accompanies mutations in genes such as KRAS, BRAF, PTEN, AKT1, all of which are components of the PI3K signaling pathway or MAPK signaling pathway (Wang and Pan, 2022; Mirzapoor Abbasabadi et al., 2023). The interaction of these signaling pathways affects the response of colorectal cancer to PI3K inhibitors.

The impact of PIK3CA gene mutations on the sensitivity to PI3K inhibitors seems to be evident, as PIK3CA gene mutations are direct targets of PI3K inhibitors. Generally, colorectal cancer with PIK3CA mutations is more sensitive to PI3K inhibitors than wild-type colorectal cancer (Voutsadakis, 2022). However, not all PIK3CA mutations will increase sensitivity to PI3K inhibitors; some studies have shown that some secondary mutations in PIK3CA, such as the W780 and Q859 mutations, actually drive resistance to certain PI3K inhibitors (Varkaris et al., 2024). The type and number of PIK3CA mutations also affect the response to PI3K inhibitors. For example, some colorectal cancer cell lines have two PIK3CA mutations, which are located on the same chromosome (referred to as cis double mutations), leading to extreme activation of the PI3K signaling pathway, making the cells more sensitive to PI3K inhibitors (Vasan et al., 2019).

mTOR is a downstream molecule of the PI3K/AKT signaling pathway, which plays an important role in regulating processes such as cell proliferation, metabolism, autophagy, and angiogenesis (Yu et al., 2022). mTOR inhibitors are a class of small molecule drugs that target the mTOR molecule, and a number of mTOR inhibitors such as everolimus and rapamycin have been investigated in clinical trials (Deng et al., 2021; Liu et al., 2022b). mTOR inhibitors are mainly used in colorectal cancer patients with PIK3CA mutations, in combination with chemotherapy or other drugs, in order to improve efficacy and overcome resistance (Fricke et al., 2019).

It is widely believed that the impact of PIK3CA gene mutations on the sensitivity to mTOR inhibitors may be positive. Some studies have shown that PIK3CA gene mutations can increase the sensitivity of colorectal cancer to mTOR inhibitors. For example, one study found that activation of the PI3K pathway due to PIK3CA gene mutations indeed leads to sensitivity to the mTOR inhibitor everolimus (Di Nicolantonio et al., 2010).

In conclusion, mutations in the PIK3CA gene have a strong effect on PI3K inhibitors as well as mTOR inhibitors, but this effect is not singular, it is modulated by a number of factors, including the characteristics of the mutation, the type of inhibitor, and the status of other signal pathways. Furthermore, in addressing adaptive resistance that arises in monotherapy with PI3K/AKT pathway inhibitors, the combined use of PI3K and mTOR inhibitors may provide an effective strategy to overcome resistance and improve therapeutic efficacy.

### 5.2 Influence and mechanism of PIK3CA gene mutation on immunotherapy in colorectal cancer

Immunotherapy is a treatment method that utilizes the body’s own immune system to identify and eliminate tumor cells (Finck et al., 2022). Currently, there are mainly two types of immunotherapies for colorectal cancer. One type is the use of immune checkpoint inhibitors, such as anti-PD-1, anti-PD-L1, and anti-CTLA-4, while the other type includes vaccines or cell therapies targeting tumor-associated antigens (Nalli et al., 2021). Both of these types of immunotherapies are closely related to PIK3CA gene mutations, which are discussed below (Figure 2).

#### 5.2.1 Influence and mechanism of PIK3CA gene mutation on immune checkpoint inhibitors

Immune checkpoints are molecular mechanisms that regulate the immune response by inhibiting the activation and proliferation of T cells, thereby maintaining immune tolerance and balance (Li et al., 2019). However, tumor cells can evade the immune system’s attack by utilizing immune checkpoints, leading to the failure of immunotherapy. Therefore, immune checkpoint inhibitors are treatments that block immune checkpoint molecules, restore the function of T cells, and enhance the immune system’s ability to kill tumor cells (Ganesh et al., 2019). Currently, there are several immune checkpoint inhibitors under clinical trials for colorectal cancer, such as the anti-PD-1 antibodies pembrolizumab and nivolumab (Overman et al., 2017; Le et al., 2020), the anti-PD-L1 antibodies atezolizumab and pembrolizumab (O'Neil et al., 2017; Winer et al., 2019), and the anti-CTLA-4 antibody ipilimumab (Winer et al., 2019).

The impact of PIK3CA gene mutations on the response and tolerance to immune checkpoint inhibitors may be positive. Some studies have shown that PIK3CA gene mutations can increase the sensitivity of colorectal cancer to immunotherapy. For example, one study found that PIK3CA gene mutations activate the PI3K pathway, which is associated with the overexpression of CD274 (PD-L1) in colorectal tumor tissues, supporting the role of PI3K signaling in the upregulation of CD274 (Ugai et al., 2021). Similarly, Ahn et al. (2021) reached the same conclusion through next-generation sequencing and immunohistochemistry. Another mechanism of sensitivity to immune checkpoint inhibitors caused by PIK3CA gene mutations is that they can increase the TMB, resulting in more neoantigens and enhancing the immunogenicity of the tumor to increase sensitivity to immune checkpoint inhibitors (Westcott et al., 2023). Some studies have shown that the TMB of colorectal cancer patients with PIK3CA gene mutations is significantly higher than that of patients with wild-type PIK3CA (Voutsadakis, 2022; Voutsadakis, 2021). These results suggest that PIK3CA gene mutations can enhance the sensitivity of tumors to immune checkpoint inhibitors by increasing TMB and enhancing the immunogenicity of the tumor. Therefore, PIK3CA gene mutations may be a predictive factor for immune checkpoint inhibitors and can be used to screen colorectal cancer patients suitable for immunotherapy.

#### 5.2.2 Impact and mechanism of PIK3CA gene mutation on tumor-associated antigen vaccines or cell therapy

Tumor-associated antigens refer to antigens expressed by tumor cells that can be recognized and cleared by the immune system, including tumor-specific antigens and tumor-associated antigens (Wagner et al., 2018). Vaccines or cell therapy targeting tumor-associated antigens are a treatment method that utilizes tumor-associated antigens to activate or enhance the immune system’s attack on tumors (Wagner et al., 2018). Currently, various tumor-associated antigen vaccines or cell therapies have entered clinical trials, such as chimeric antigen receptor (CAR-T) cell therapy, autologous vaccine OncoVAX therapy, and tumor-infiltrating lymphocyte (TIL) therapy, etc. (Zhang et al., 2019; Fan et al., 2021).

The impact and mechanism of PIK3CA gene mutation on tumor-associated antigen vaccines or cell therapy in colorectal cancer mainly involve several aspects. Firstly, PIK3CA gene mutation itself is a new tumor antigen that can be recognized and attacked by the immune system (Chen et al., 2020). Studies have found that cationic micelle delivery of multi-epitope candidate vaccines derived from tumor-associated antigens (including PIK3CA) can lead to regression of established CT26 colon tumors in mice (Sabzehei et al., 2023). Therefore, the development of vaccines or cell therapy targeting PIK3CA gene mutation may also have certain therapeutic effects. Secondly, PIK3CA gene mutation may affect the types and quantities of other tumor-associated antigens expressed by tumor cells, thereby affecting the efficacy of vaccines or cell therapy. For example, PIK3CA gene mutation can activate the PI3K/AKT signaling pathway, increase the mutational load and generation of neoantigens in tumor cells, thereby increasing the response rate of vaccines or cell therapy (Westcott et al., 2023). Lastly, the influence of PIK3CA gene mutations on the tumor microenvironment (TME) includes both vascular and immune modulation. On one hand, PIK3CA mutations activate the PI3K/AKT signaling pathway, which promotes angiogenesis, resulting in increased tumor vascularization. This can reduce the efficiency of vaccine or cell-based therapies by limiting their targeting and penetrance due to enhanced tumor infiltration and metastasis (Liu et al., 2009). On the other hand, PIK3CA mutations alter the immune landscape of the TME by affecting the production of cytokines and chemokines. This results in changes to the quantity, type, and activity of tumor-infiltrating lymphocytes (TILs), which are critical for an effective immune response. For instance, a shift in the balance of pro-inflammatory *versus* anti-inflammatory cytokines may impair the activation and persistence of immune cells, thereby reducing the effectiveness and durability of vaccines or cell therapies targeting the tumor (Nosho et al., 2010).

In conclusion, the impact and mechanism of PIK3CA gene mutation on tumor-associated antigen vaccines or cell therapy in colorectal cancer is complex, involving multiple aspects such as tumor cells, immune cells, signaling pathways, and the tumor microenvironment.

## 6 Future directions of PIK3CA gene mutations in individualized treatment of colorectal cancer

The role of PIK3CA gene mutations in colorectal cancer and the choice of treatment strategies is an important and challenging research area with significant clinical implications. With a deeper understanding of the molecular mechanisms and clinical impact of PIK3CA gene mutations, as well as the development and application of targeted drugs against PIK3CA, more precise and effective treatment options have been provided for colorectal cancer patients. However, there are still unresolved issues and difficulties, such as the heterogeneity and diversity of PIK3CA gene mutations, resistance and toxic side effects of PIK3CA inhibitors, and the lack of uniform and accurate detection methods and standards for PIK3CA gene mutations. Therefore, in order to better utilize PIK3CA gene mutations as indicators for guiding and evaluating individualized treatment for colorectal cancer, it is necessary to conduct further research and exploration in the following areas.

First, the development of more effective and specific PIK3CA inhibitors. Currently, several inhibitors targeting PIK3CA have entered clinical trials or are in use, such as pictilisib, apelisib, and buparlisib, which are primarily selective inhibitors of the p110α subtype and have some efficacy against the most common exons 9 and 20 PIK3CA mutations in colorectal cancer (André et al., 2019; Bardia et al., 2020; Sarker et al., 2015). However, these inhibitors also have limitations, such as insufficient sensitivity to other PIK3CA mutations, unclear effects on other signaling pathways, and greater toxicity to normal cells. Therefore, there is a need to develop more specific and potent PIK3CA inhibitors to cover a broader spectrum of PIK3CA mutations and more precisely inhibit key nodes of the PI3K signaling pathway while reducing damage to normal cells.

Next, explore more rational and flexible drug combinations or sequential treatment regimens. Due to the heterogeneity and diversity of PIK3CA gene mutations (Wang et al., 2018b), as well as the resistance and toxic side effects of PIK3CA inhibitors (Mishra et al., 2021), single-agent PIK3CA inhibitor therapy may be difficult to achieve the desired effect. Therefore, it is necessary to explore combination or sequential treatment regimens with other drugs to improve treatment efficacy and reduce toxicity. Currently, some studies have shown that the combination or sequential use of PIK3CA inhibitors with anti-EGFR monoclonal antibodies, MEK inhibitors, mTOR inhibitors, immunotherapy, etc., can enhance anti-tumor effects, prolong progression-free survival, or overcome resistance (Napolitano et al., 2023; Kuboki et al., 2024; Goodwin et al., 2020). However, these studies still have some limitations, such as small sample size, imperfect study design, inconsistent drug doses and regimens, and inconsistent methods and standards for PIK3CA mutation detection. Therefore, more clinical trials are needed to determine the best drug combinations or sequential treatment regimens, as well as the most suitable patient subgroups.

Finally, optimize and standardize the detection methods and standards for PIK3CA gene mutations. The detection of PIK3CA gene mutations is the prerequisite and basis for personalized treatment of colorectal cancer, however, there is still a lack of a unified and accurate detection method and standard. Currently, commonly used methods for detecting PIK3CA gene mutations include real-time PCR, digital droplet PCR, Sanger sequencing, and next-generation sequencing (NGS) (Fu et al., 2021; Alizadeh-Sedigh et al., 2022; Wang et al., 2019). These methods have their own advantages and disadvantages, such as sensitivity, specificity, coverage, cost, and time. In addition, different detection methods may lead to different results, such as differences in false negatives, false positives, mutation types and frequencies. Therefore, it is necessary to optimize and standardize the detection methods and standards for PIK3CA gene mutations to improve the accuracy and reliability of detection, as well as the consistency and comparability between different detection methods.

## 7 Conclusion

PIK3CA gene mutation is one of the most common molecular alterations in colorectal cancer. It plays an important role and significance in the occurrence and development, clinical characteristics and prognosis, targeted therapy, and the effects and mechanisms of immunotherapy in colorectal cancer. This review summarized the detection methods and standards for PIK3CA gene mutations, analyzed the correlation and carcinogenicity of PIK3CA gene mutations in colorectal cancer, discussed the impact and mechanism of PIK3CA gene mutations on the treatment of colorectal cancer, as well as the future directions of PIK3CA gene mutations in individualized treatment of colorectal cancer. Table 1 summarizes the current clinical applications of PIK3CA mutations in CRC, highlighting their diagnostic, prognostic, and therapeutic relevance.

**TABLE 1:** Clinical applications of PIK3CA mutations in colorectal cancer.

Mutation type	Clinical use	Therapies/Drugs	Prognostic value	References
PIK3CA	Prognostic marker, used for treatment selection, particularly in advanced CRC	PI3K inhibitors (e.g., alpelisib), combination therapies with mTOR or EGFR inhibitors	Variable (worse with exon 20 mutations, better with exon 9 mutations)	Dekker et al. (2019), Yan et al. (2022), Gustavsson et al. (2015)
Exon 9 mutations	Resistance to anti-EGFR therapy is generally not observed with exon 9 mutations	Cetuximab, panitumumab (for KRAS/NRAS wild-type)	Associated with better outcomes compared to exon 20 mutations	Dekker et al. (2019), Gupta et al. (2019)
Exon 20 mutations	Associated with resistance to anti-EGFR therapy, considered in targeted treatment decision-making	PI3K inhibitors, mTOR inhibitors, combination therapies	Worse prognosis, linked to advanced disease and higher recurrence	Dekker et al. (2019), Gustavsson et al. (2015), Zhou et al. (2018)
PIK3CA + PD-L1	Potential use in immunotherapy selection, linked to enhanced PD-L1 expression and TMB	Immune checkpoint inhibitors (e.g., pembrolizumab)	Possibly improved immunotherapy response	Amirouchene-Angelozzi et al. (2017), Graham and Sottoriva (2017)

However, this review also has some limitations and shortcomings that need further research and resolution. Firstly, the detection methods and standards for PIK3CA gene mutations have not been unified and standardized, and different detection platforms and technologies may lead to differences and incomparability in results. Secondly, the function and mechanism of PIK3CA gene mutations have not been fully revealed, especially in terms of their interaction and synergistic effects with other molecular alterations, as well as their relationship with the tumor microenvironment. Thirdly, the impact and mechanism of PIK3CA gene mutations on the treatment of colorectal cancer are not yet clear, especially in terms of their impact on immunotherapy. Fourthly, the application of PIK3CA gene mutations in individualized treatment of colorectal cancer is still in its early stages, and more clinical trials and evidence are needed to verify its effectiveness and safety, as well as to optimize its treatment regimens and guiding principles.

We hope for more collaboration and innovation in basic and clinical research to improve the understanding and utilization of PIK3CA gene mutations, and thus promote the development and progress of individualized treatment for colorectal cancer, bringing better prognosis and quality of life to colorectal cancer patients.
